# Diversity of the longhorned beetles (Coleoptera: Cerambycidae) from Cuatro Ciénegas Basin, Coahuila, Mexico

**DOI:** 10.3897/BDJ.8.e54495

**Published:** 2020-07-16

**Authors:** Oscar Pérez-Flores, Víctor H. Toledo-Hernández

**Affiliations:** 1 Colección Nacional de Insectos, Instituto de Biología, Universidad Nacional Autónoma de México, Delegación Coyoacán, Mexico City, Mexico Colección Nacional de Insectos, Instituto de Biología, Universidad Nacional Autónoma de México Delegación Coyoacán, Mexico City Mexico; 2 Centro de Investigación en Biodiversidad y Conservación (CIByC), Universidad Autónoma del Estado de Morelos, Cuernavaca, Morelos, Mexico Centro de Investigación en Biodiversidad y Conservación (CIByC), Universidad Autónoma del Estado de Morelos Cuernavaca, Morelos Mexico

**Keywords:** Cerambycid beetles, Chihuahuan desert, distribution, North America.

## Abstract

**Background:**

Cerambycidae is one the most diverse families in the order Coleoptera with more than 37,000 species described in all continents. Cerambicyd beetles have a worldwide distribution from sea level to montane sites. In Mexico, more than 1,600 species have been recorded. Nevertheless, the diversity and distribution of this family in the Mexican deserts is poorly known.

**New information:**

A first checklist of Cerambycidae from seven localities of Cuatro Ciénegas Basin is presented. This study is the result of sampling carried out between 2009 to 2013. Some material from other collections is also included. The species list includes four subfamilies, 13 tribes, 32 genera and 37 species, from which 13 are new records for the state of Coahuila and three species represent new records for Mexico. These results highlight the paucity of knowledge of insects in the deserts of Mexico.

## Introduction

Cerambycidae comprise one of the largest families of Coleoptera, represented by more than 37,000 species, described in more than 5,300 genera from eight subfamilies (Monné et al. 2017, Tavakilian and Chevillotte 2020). Cerambycid beetles are distributed worldwide wherever their hosts plants are found from sea level to montane sites as high as 4,200 m elevation (Bezark et al. 2013). Due to its ecological, biological and evolutionary importance, this family has been studied extensively. The diversity of the Cerambycidae in Mexico was summarised by Noguera (2014). However, knowledge of the diversity and distribution of the family Cerambycidae in the Mexican deserts is poor, with just some isolated records (Bezark 2020).

**Figure 1.**
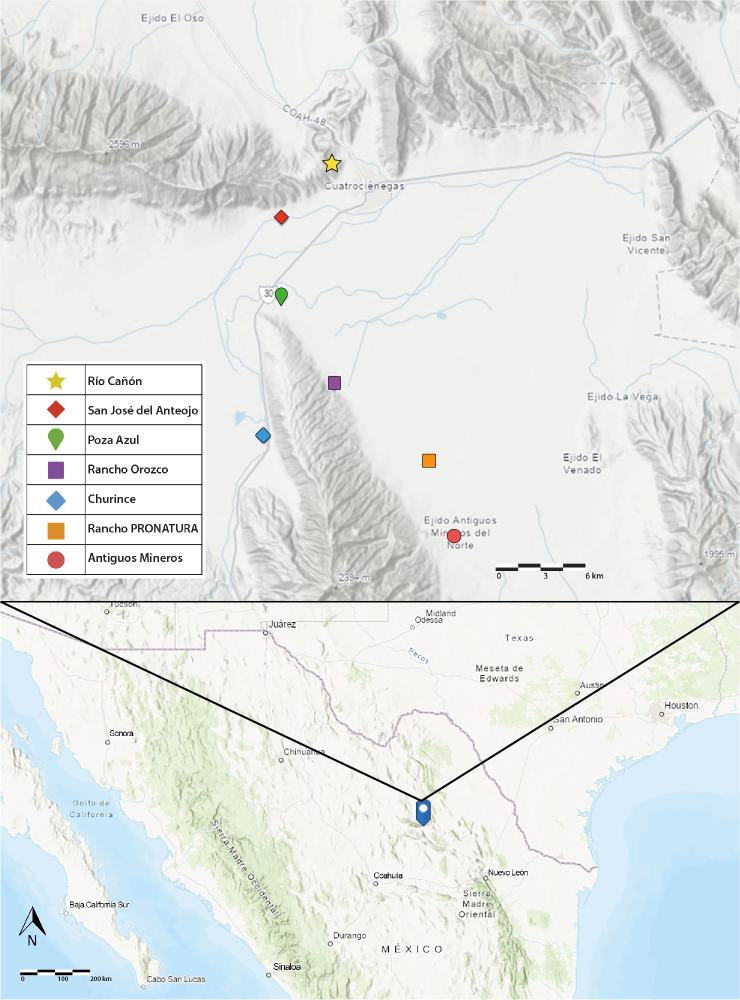
Map showing sampling localities from Cuatro Ciénegas Basin, Coahuila, Mexico.

The Cuatro Ciénegas Basin (CCB) (Souza et al. 2012) is located at the south-eastern portion of the Chihuahuan Desert (Fig. 1). With more than 450,000 km^2^, it is the largest desert in North America. This, along with its high biological diversity, makes it one of the most important deserts in the world (Morafka 1977). The CCB has a unique geological and biological history which dates from at least 40 mya in the Middle Eocene. At this point the region was isolated from the Atlantic Ocean by the rise of the Sierra Madre Oriental (Álvarez and Ojeda 2019). The subterranean water, which currently emerges, forms ponds and streams that distinguish this region from other surrounding desert areas. This, plus the isolation of the CCB, has resulted in a high degree of endemism in some taxonomic groups (Souza et al. 2012).

The fauna that occurs in the CCB is a mixture of species with Neotropical and Nearctic biogeographic origins or affinities. This important region of transition can represent the distributional limits of both groups (Souza et al. 2012, Álvarez and Ojeda 2019). This work is a result of a study of diverse taxonomic groups in the CCB (Álvarez and Ojeda 2019), undertaken for the purpose of providing a better understanding of its local diversity. There are no published records of cerambycids from this region. This study is the first approach that aims to contribute to the general knowledge of this group in the Mexican deserts.

## Materials and methods

The dominant habitat in CCB is desertic shrub of the microphyle and rosetophyle variants, which include sotol, mesquite, secondary grassland and semi-aquatic vegetation associated with ponds. The climate is desertic dry (BW) (García 1964) with three well-defined seasons: rainy, cold dry and hot dry. All localities that were selected have superficial water (except Río Cañón) and included all habitats present in CCB. Most habitats had some degree of perturbation due to different issues such as cattle grazing, agriculture and tourism. The localities were the following: (1) Churince (26°50'30.3"N, 102°08'9.9"W), (2) Rancho PRONATURA (26°49'38.9"N, 102°01'24.4"W), (3) Rancho Orozco (26°52'18.3"N, 102°05'16.8"W), (4) Antiguos Mineros (26°46'57.7"N, 102°00'20.2"W), (5) Poza Azul (26°55'21.2"N, 102°72'3.6"W), (6) Río Cañón (27°00'33.7"N, 102°04'42.3"W) and (7) San José del Anteojo (26°58'11.5"N, 102°07'26.4"W) (Fig. 1). The distribution map was made using ArcGIS server (Bader 2005).

Fourteen field trips were performed during 2012 and 2013. Records from sporadic trips in 2009 and 2010 are also included. Sampling totalled 84 collecting days with at least three people collecting each day (09:00 to 16:00 h). Different sampling techniques (direct and indirect) were applied: by hand, sweep net and light traps. Cerambycid beetles were identified to species level with specialised literature (Chemsak 1991, Chemsak and Linsley 1965, Chemsak and Noguera 2005, Giesbert and Chemsak 1989, Giesbert and Chemsak 1993, Grzymala and Miller 2013, Linsley 1963, Linsley 1964, Linsley and Chemsak 1972, Noguera 2002, Santos-Silva 2007, Santos-Silva et al. 2016, Turnbow and Thomas 2002) and by comparison with identified specimens from the National Collection of Insects of the Biology Institute, UNAM. All specimens were deposited in the Insect Collection of the Zoological Museum “Alfonso L. Herrera” of the Faculty of Sciences, UNAM. The National Collection of Insects (CNIN) was revised and all the records belonging with CCB were included (Suppl. material 1).

## Checklists

### List of Cerambycidae from Cuatro Ciénegas Basin. The taxonomy is based on Bezark (2020)

#### Prioninae

4D8CD432-5604-5F63-8993-BBBAA06A366C

#### Prionini

DB8C4B09-EB44-545E-981D-C2D5DED5C975

#### Derobrachus hovorei

Santos-Silva, 2007

24BCE4E7-8CAA-5B7B-836B-8CD83B7DEA9B

##### Materials

- **Type status:** Other material. **Occurrence:** recordedBy: Marysol Trujano and Uri García; **Location:** country: Mexico; stateProvince: Coahuila; municipality: Cuatro Ciénegas; locality: Rancho PRONATURA; verbatimCoordinates: 26°49'38.9"N, 102°01'24.4"W; **Event:** samplingProtocol: Light trap; eventDate: 7-Jul-13; habitat: Desertic shrub; **Record Level:** collectionCode: MZ-FC_CCB01; basisOfRecord: Preserved Specimen
- **Type status:** Other material. **Occurrence:** recordedBy: Marysol Trujano and Uri García; **Location:** country: Mexico; stateProvince: Coahuila; municipality: Cuatro Ciénegas; locality: Churince; verbatimCoordinates: 26°50'30.3"N, 102°08'9.9"W; **Event:** samplingProtocol: Light trap; eventDate: 20-Apr-12; habitat: Desertic shrub; **Record Level:** collectionCode: MZ-FC_CCB02; basisOfRecord: Preserved Specimen

##### Ecological interactions

###### Host of

*Cercidium microphyllum* Rose and I. M. Johnston (Caesalpiniaceae), *Quercus* sp. (Fagaceae), *Prosopis juliflora* (Swartz) de Candolle (Mimosaceae), *Morus rubra* Linnaeus (Moraceae), *Citrus* sp. (Rutaceae), *Populus* sp. (Salicaceae), *Ulmus* sp. (Ulmaceae), *Vitis* sp. (Vitaceae). (Monné 2020)

##### Distribution

USA: Arizona, California, Nevada, New Mexico, Texas; MEXICO: Chihuahua, Coahuila, Durango, Nuevo León, Sonora, Tamaulipas.

##### Notes

MZ-FC

#### Prionus (Prionus) poultoni

Lameere, 1912

E1855807-1E73-5C32-A050-BDFBC56DF9C3

##### Materials

- **Type status:** Other material. **Occurrence:** recordedBy: Antonio Castro; **Location:** country: Mexico; stateProvince: Coahuila; municipality: Cuatro Ciénegas; locality: La Becerra; **Event:** samplingProtocol: By hand; eventDate: 15-Aug-14; habitat: Desertic shrub; **Record Level:** collectionCode: CNIN_CCB01; basisOfRecord: Preserved Specimen

##### Distribution

MEXICO: Chihuahua, Coahuila, Nuevo León, Oaxaca, Veracruz.

##### Notes

CNIN

#### Lepturinae

4DFDDBE9-EDB0-50F8-959B-B46ABFBED673

#### Lepturini

800581EC-2887-54C7-8450-26C8CDBDB630

#### Meloemorpha aliena

(Bates, 1880)

89DEC639-DC22-5DEE-A8CC-AA05600B63DD

##### Materials

- **Type status:** Other material. **Occurrence:** recordedBy: Oscar Pérez; **Location:** country: Mexico; stateProvince: Coahuila; municipality: Cuatro Ciénegas; locality: Churince; verbatimCoordinates: 26°50'19.36", 102°09'04.38"W; **Event:** samplingProtocol: By hand; eventDate: 21-Jul-15; habitat: Desertic shrub; **Record Level:** collectionCode: CNIN_CCB02; basisOfRecord: Preserved Specimen
- **Type status:** Other material. **Occurrence:** recordedBy: Oscar Pérez; **Location:** country: Mexico; stateProvince: Coahuila; municipality: Cuatro Ciénegas; locality: Churince; verbatimCoordinates: 26°50'19.36"N,102°09'04.38"W; **Event:** samplingProtocol: By hand; eventDate: 21-Jul-15; habitat: Desertic shrub; **Record Level:** collectionCode: CNIN_CCB03; basisOfRecord: Preserved Specimen

##### Ecological interactions

###### Host of

*Pinus pseudostrobus* Lindley, *P. rudis* Endlicher (Pinaceae). (Monné 2020)

##### Distribution

MEXICO: Ciudad de México, Coahuila (new state record), Guerrero, Hidalgo, México, Michoacán, Morelos, Nuevo León, Veracruz; GUATEMALA.

##### Notes

CNIN

#### Typocerus sinuatus

(Newman, 1841)

1F1A1F01-C83C-530C-992C-BD7F24102EB2

##### Materials

- **Type status:** Other material. **Occurrence:** recordedBy: Marysol Trujano and Uri García; **Location:** country: Mexico; stateProvince: Coahuila; municipality: Cuatro Ciénegas; locality: Rancho Orozco; verbatimCoordinates: 26°52'18.3"N, 102°05'16.8"W; **Event:** samplingProtocol: Sweeping; eventDate: 12-Apr-12; habitat: Desertic shrub; **Record Level:** collectionCode: MZ-FC_CCB03; basisOfRecord: Preserved Specimen

##### Distribution

USA: Colorado, New Mexico; MEXICO: Coahuila (new country record).

##### Notes

MZ-FC

#### Cerambycinae

202A7E8D-8CDB-5ECA-8E31-F9602095F2AA

#### Bothriospilini

E43D2A41-8AE6-5252-95FA-19E21805923B

#### Knulliana cincta cincta

(Drury, 1773)

7CBFAC0A-5602-586B-852C-657690B8C810

##### Materials

- **Type status:** Other material. **Occurrence:** recordedBy: Marysol Trujano and Uri García; **Location:** country: Mexico; stateProvince: Coahuila; municipality: Cuatro Ciénegas; locality: San José del Anteojo; verbatimCoordinates: 26°58'11.5"N, 102°07'26.4"W; **Event:** samplingProtocol: By hand; eventDate: 23-May-12; habitat: Desertic shrub; **Record Level:** collectionCode: MZ-FC_CCB04; basisOfRecord: Preserved Specimen

##### Distribution

USA: Texas; MEXICO: Coahuila (new state record), Nuevo León, Sonora.

##### Notes

MZ-FC

#### Clytini

501CB3EE-6776-507B-9075-4014E4B80D66

#### Megacyllene antennata

(White, 1855)

0EC4012A-9CE3-5E4C-84AE-E3961E6813CB

##### Materials

- **Type status:** Other material. **Occurrence:** recordedBy: Marysol Trujano and Uri García; **Location:** country: Mexico; stateProvince: Coahuila; municipality: Cuatro Ciénegas; locality: Churince; verbatimCoordinates: 26°50'30.3"N, 102°08'9.9"W; **Event:** samplingProtocol: By hand; eventDate: 24-Sep-12; habitat: Desertic shrub; **Record Level:** collectionCode: MZ-FC_CCB05; basisOfRecord: Preserved Specimen

##### Ecological interactions

###### Host of

*Celtis reticulata* Torr. (Cannabaceae), *Prosopis juliflora* (Swartz) de Candolle, *Senegalia greggii* A. Gray (Mimosaceae). (Monné 2020)

##### Distribution

USA: California, Texas; MEXICO: Baja California Norte, Coahuila (new state record), Sinaloa, Sonora.

##### Notes

MZ-FC

#### Neoclytus mucronatus mucronatus

(Fabricius, 1775)

1D40C117-4028-5A8C-9069-7D5C1C8F1C8B

##### Materials

- **Type status:** Other material. **Occurrence:** recordedBy: Rodolfo Trejo; **Location:** country: Mexico; stateProvince: Coahuila; municipality: Cuatro Ciénegas; locality: Poza Azul; **Event:** samplingProtocol: By hand; eventDate: 17-Oct-16; habitat: Desertic shrub; **Record Level:** collectionCode: CNIN_CCB04; basisOfRecord: Preserved Specimen
- **Type status:** Other material. **Occurrence:** recordedBy: Rodolfo Trejo; **Location:** country: Mexico; stateProvince: Coahuila; municipality: Cuatro Ciénegas; locality: Poza Azul; **Event:** samplingProtocol: By hand; eventDate: 17-Oct-16; habitat: Desertic shrub; **Record Level:** collectionCode: CNIN_CCB05; basisOfRecord: Preserved Specimen
- **Type status:** Other material. **Occurrence:** recordedBy: Rodolfo Trejo; **Location:** country: Mexico; stateProvince: Coahuila; municipality: Cuatro Ciénegas; locality: Poza Azul; **Event:** samplingProtocol: By hand; eventDate: 17-Oct-16; habitat: Desertic shrub; **Record Level:** collectionCode: CNIN_CCB06; basisOfRecord: Preserved Specimen

##### Ecological interactions

###### Host of

*Acer rubrum* Linné (Aceraceae), *Celtis laevigata* Willdnow (Cannabaceae), *Juniperus virginiana* Linné (Cupressaceae), *Diospyros virginiana* Linné (Ebenaceae), *Quercus* sp. (Fagaceae), *Carya glabra* (Miller) Sweet, *C. illinoinensis* (Wangenheim) K. Koch, *C. ovata* (Miller) K. Koch, *C. tomentosa* Nuttall (Juglandaceae), *Pinus contorta* Douglas ex Loudon, *P. resinosa* Aiton (Pinaceae), *Ulmus* sp. (Ulmaceae). (Monné 2020)

##### Distribution

USA: Texas; MEXICO: Coahuila (new country record).

##### Notes

CNIN

#### Placosternus erythropus

(Chevrolat, 1835)

A07FC776-6483-519C-B6FE-DDCD86A0B9D6

##### Materials

- **Type status:** Other material. **Occurrence:** recordedBy: Marysol Trujano and Uri García; **Location:** country: Mexico; stateProvince: Coahuila; municipality: Cuatro Ciénegas; locality: Rancho Orozco; verbatimCoordinates: 26°52'18.3"N, 102°05'16.8"W; **Event:** samplingProtocol: Sweeping; eventDate: 23-Oct-13; habitat: Desertic shrub; **Record Level:** collectionCode: MZ-FC_CCB06; basisOfRecord: Preserved Specimen
- **Type status:** Other material. **Occurrence:** recordedBy: Marysol Trujano and Uri García; **Location:** country: Mexico; stateProvince: Coahuila; municipality: Cuatro Ciénegas; locality: Rancho Orozco; verbatimCoordinates: 26°52'18.3"N, 102°05'16.8"W; **Event:** samplingProtocol: Sweeping; eventDate: 23-Oct-13; habitat: Desertic shrub; **Record Level:** collectionCode: MZ-FC_CCB07; basisOfRecord: Preserved Specimen
- **Type status:** Other material. **Occurrence:** recordedBy: Marysol Trujano and Uri García; **Location:** country: Mexico; stateProvince: Coahuila; municipality: Cuatro Ciénegas; locality: Poza Azul; verbatimCoordinates: 26°55'21.2"N, 102°72'3.6"W; **Event:** samplingProtocol: Sweeping; eventDate: 24-Aug-12; habitat: Desertic shrub; **Record Level:** collectionCode: MZ-FC_CCB08; basisOfRecord: Preserved Specimen

##### Ecological interactions

###### Host of

*Baccharis neglecta* Nuttall (Asteraceae), *Acacia hindsii* Bentham, *Prosopis dulcis* Kunth, *P. juliflora* (Swartz) de Candolle (Mimosaceae), *Condalia globosa* I. M. Johnston (Rhamnaceae), *Crataegus pubescens* Steudel, *Malus sylvestris* Miller, *Prunus capollin* Zuccarini, *P. domestica* Linné, *P. persica* (Linné) Batsch, *Pyrus communis* Willdenow (Rosaceae). (Monné 2020)

##### Distribution

USA: Texas; MEXICO: Ciudad de México, Coahuila, Durango, Guanajuato, Hidalgo, Jalisco, México, Morelos, Puebla, Sonora, Tabasco, Tamaulipas, Veracruz; GUATEMALA; COSTA RICA

##### Notes

MZ-FC

#### Tanyochraethes hololeucus

(Bates, 1892)

1EBD5748-89CB-5591-99EE-24950B2E6219

##### Materials

- **Type status:** Other material. **Occurrence:** recordedBy: Marysol Trujano and Uri García; **Location:** country: Mexico; stateProvince: Coahuila; municipality: Cuatro Ciénegas; locality: Antiguos Mineros; verbatimCoordinates: 26°46'57.7"N, 102°00'20.2"W; **Event:** samplingProtocol: By hand; eventDate: 22-Nov-12; habitat: Desertic shrub; **Record Level:** collectionCode: MZ-FC_CCB09; basisOfRecord: Preserved Specimen
- **Type status:** Other material. **Occurrence:** recordedBy: Marysol Trujano and Uri García; **Location:** country: Mexico; stateProvince: Coahuila; municipality: Cuatro Ciénegas; locality: Antiguos Mineros; verbatimCoordinates: 26°46'57.7"N, 102°00'20.2"W; **Event:** samplingProtocol: By hand; eventDate: 22-Nov-12; habitat: Desertic shrub; **Record Level:** collectionCode: MZ-FC_CCB10; basisOfRecord: Preserved Specimen

##### Distribution

MEXICO: Coahuila, Durango, Tamaulipas.

##### Notes

MZ-FC

#### Eburiini

DF5DB34C-666E-5069-8E13-DFA998CA6C55

#### Eburia juanitae

Chemsak and Linsley, 1970

2DF1D207-2D90-50FF-9A59-D230D3FF6414

##### Materials

- **Type status:** Other material. **Occurrence:** recordedBy: Marysol Trujano and Uri García; **Location:** country: Mexico; stateProvince: Coahuila; municipality: Cuatro Ciénegas; locality: Río Cañón; verbatimCoordinates: 27°00'33.7"N, 102°04'42.3"W; **Event:** samplingProtocol: Sweeping; eventDate: 7-Jul-10; habitat: Desertic shrub; **Record Level:** collectionCode: MZ-FC_CCB11; basisOfRecord: Preserved Specimen

##### Distribution

MEXICO: Coahuila, Jalisco, Morelos, Oaxaca, Sinaloa.

##### Notes

MZ-FC

#### Eburia maccartyi

Noguera, 2002

0830385C-EBA0-5AF8-BCB0-DC87C941962B

##### Materials

- **Type status:** Other material. **Occurrence:** recordedBy: Oscar Pérez; **Location:** country: Mexico; stateProvince: Coahuila; municipality: Cuatro Ciénegas; locality: Poza Azul; verbatimCoordinates: 26°55'28.03"N, 102°06'56.46"W; **Event:** samplingProtocol: Sweeping; eventDate: 29-Jul-15; habitat: Desertic shrub; **Record Level:** collectionCode: CNIN_CCB07; basisOfRecord: Preserved Specimen

##### Distribution

MEXICO: Coahuila, Colima, Guanajuato, Guerrero, Jalisco, Nayarit, Oaxaca.

##### Notes

CNIN

#### Eburia paraegrota

Chemsak and Linsley, 1973

0D6FE446-8F02-5FBB-9581-9FC15EFC4338

##### Materials

- **Type status:** Other material. **Occurrence:** recordedBy: Marysol Trujano and Uri García; **Location:** country: Mexico; stateProvince: Coahuila; municipality: Cuatro Ciénegas; locality: San José del Anteojo; verbatimCoordinates: 26°58'11.5"N, 102°07'26.4"W; **Event:** samplingProtocol: By hand; eventDate: 23-Aug-09; habitat: Desertic shrub; **Record Level:** collectionCode: MZ-FC_CCB12; basisOfRecord: Preserved Specimen
- **Type status:** Other material. **Occurrence:** recordedBy: Marysol Trujano and Uri García; **Location:** country: Mexico; stateProvince: Coahuila; municipality: Cuatro Ciénegas; locality: San José del Anteojo; verbatimCoordinates: 26°58'11.5"N, 102°07'26.4"W; **Event:** samplingProtocol: By hand; eventDate: 23-Aug-09; habitat: Desertic shrub; **Record Level:** collectionCode: MZ-FC_CCB13; basisOfRecord: Preserved Specimen
- **Type status:** Other material. **Occurrence:** recordedBy: Marysol Trujano and Uri García; **Location:** country: Mexico; stateProvince: Coahuila; municipality: Cuatro Ciénegas; locality: San José del Anteojo; verbatimCoordinates: 26°58'11.5"N, 102°07'26.4"W; **Event:** samplingProtocol: By hand; eventDate: 23-Aug-09; habitat: Desertic shrub; **Record Level:** collectionCode: MZ-FC_CCB14; basisOfRecord: Preserved Specimen

##### Distribution

MEXICO: Coahuila, Colima, Jalisco, Sinaloa.

##### Notes

MZ-FC

#### Elaphidiini

C63B2EE4-72B3-50A6-8747-FD5F3A60D991

#### Aneflus (Aneflus) obscurus

(LeConte, 1873)

B2A10634-D6FF-58D4-9CC9-332C7EB23A8D

##### Materials

- **Type status:** Other material. **Occurrence:** recordedBy: Marysol Trujano and Uri García; **Location:** country: Mexico; stateProvince: Coahuila; municipality: Cuatro Ciénegas; locality: Río Cañón; verbatimCoordinates: 27°00'33.7"N, 102°04'42.3"W; **Event:** samplingProtocol: Light trap; eventDate: 4-Jul-13; habitat: Desertic shrub; **Record Level:** collectionCode: MZ-FC_CCB15; basisOfRecord: Preserved Specimen
- **Type status:** Other material. **Occurrence:** recordedBy: Marysol Trujano and Uri García; **Location:** country: Mexico; stateProvince: Coahuila; municipality: Cuatro Ciénegas; locality: Río Cañón; verbatimCoordinates: 27°00'33.7"N, 102°04'42.3"W; **Event:** samplingProtocol: Light trap; eventDate: 4-Jul-13; habitat: Desertic shrub; **Record Level:** collectionCode: MZ-FC_CCB16; basisOfRecord: Preserved Specimen

##### Ecological interactions

###### Host of

*Acacia* sp. (Mimosaceae). (Monné 2020)

##### Distribution

USA: Arizona, Texas; MEXICO: Chihuahua, Coahuila.

##### Notes

MZ-FC

#### Aneflus (Aneflus) prolixus insoletus

Chemsak and Linsley, 1963

C73A8751-1908-5EF7-8FD8-20D19AB52904

##### Materials

- **Type status:** Other material. **Occurrence:** recordedBy: Marysol Trujano and Uri García; **Location:** country: Mexico; stateProvince: Coahuila; municipality: Cuatro Ciénegas; locality: Churince; verbatimCoordinates: 26°50'30.3"N, 102°08'9.9"W; **Event:** samplingProtocol: Light trap; eventDate: 20-Aug-12; habitat: Desertic shrub; **Record Level:** collectionCode: MZ-FC_CCB17; basisOfRecord: Preserved Specimen

##### Ecological interactions

###### Host of

*Acacia berlandieri* Bentham (Mimosaceae). (Monné 2020)

##### Distribution

USA: Texas; MEXICO: Chihuahua, Coahuila, Durango, Hidalgo, San Luis Potosí, Tamaulipas.

##### Notes

MZ-FC

#### Aneflomorpha rectilinea rectilinea

Casey, 1924

45DA8767-235B-57C8-8D9D-39D7468C44B2

##### Materials

- **Type status:** Other material. **Occurrence:** recordedBy: Marysol Trujano and Uri García; **Location:** country: Mexico; stateProvince: Coahuila; municipality: Cuatro Ciénegas; locality: Rancho PRONATURA; verbatimCoordinates: 26°49'38.9"N, 102°01'24.4"W; **Event:** samplingProtocol: Light trap; eventDate: 6-Jul-13; habitat: Desertic shrub; **Record Level:** collectionCode: MZ-FC_CCB18; basisOfRecord: Preserved Specimen
- **Type status:** Other material. **Occurrence:** recordedBy: Marysol Trujano and Uri García; **Location:** country: Mexico; stateProvince: Coahuila; municipality: Cuatro Ciénegas; locality: Rancho PRONATURA; verbatimCoordinates: 26°49'38.9"N, 102°01'24.4"W; **Event:** samplingProtocol: Light trap; eventDate: 7-Jul-13; habitat: Desertic shrub; **Record Level:** collectionCode: MZ-FC_CCB19; basisOfRecord: Preserved Specimen

##### Ecological interactions

###### Host of

*Rhus aromatica* Aitchison (Anacardiaceae), *Baccharis sarothroides* A. Gray (Asteraceae), *Quercus* sp. (Fagaceae). (Monné 2020)

##### Distribution

USA: Arizona, New Mexico, Texas; MEXICO: Baja California Norte, Coahuila (new state record), Jalisco, Sinaloa.

##### Notes

MZ-FC

#### Aneflomorpha werneri

Chemsak, 1962

CA381AFA-DEA2-5FCF-A6A6-8443D8431965

##### Materials

- **Type status:** Other material. **Occurrence:** recordedBy: Marysol Trujano and Uri García; **Location:** country: Mexico; stateProvince: Coahuila; municipality: Cuatro Ciénegas; locality: Poza Azul; verbatimCoordinates: 26°55'21.2"N, 102°72'3.6"W; **Event:** samplingProtocol: Light trap; eventDate: 12-Apr-12; habitat: Desertic shrub; **Record Level:** collectionCode: MZ-FC_CCB20; basisOfRecord: Preserved Specimen

##### Distribution

USA: Texas; MEXICO: Coahuila (new country record).

##### Notes

MZ-FC

#### Anelaphus moestus moestus

(LeConte, 1854)

6FF65474-F8CA-5090-B8D2-D83D3255C087

##### Materials

- **Type status:** Other material. **Occurrence:** recordedBy: Marysol Trujano and Uri García; **Location:** country: Mexico; stateProvince: Coahuila; municipality: Cuatro Ciénegas; locality: Antiguos Mineros; verbatimCoordinates: 26°46'57.7"N, 102°00'20.2"W; **Event:** samplingProtocol: Light trap; eventDate: 21-Jun-09; habitat: Desertic shrub; **Record Level:** collectionCode: MZ-FC_CCB21; basisOfRecord: Preserved Specimen

##### Ecological interactions

###### Host of

*Rhus aromatica* Aitchison (Anacardiaceae), *Grindelia* sp. (Asteraceae), *Celtis laevigata* Willdenow, *C. lindheimeri* Engelmann, *C. reticulata* Torrey (Cannabaceae), *Sophora secundiflora* (Ortega) de Candolle (Fabaceae), *Quercus* sp. (Fagaceae), *Juglans nigra* Linnaeus (Juglandaceae), *Pithecellobium flexicaule* (Bentham) Coulter, *Prosopis juliflora* (Swartz) de Candolle, *Vachellia rigidula* (Bentham) Seigler and Ebinger, (Mimosaceae), *Morus rubra* Linnaeus (Moraceae), *Pinus cembroides* Zuccarini (Pinaceae), *Zanthoxylum clava-herculis* Linnaeus (Rutaceae). (Monné 2020)

##### Distribution

USA: Arizona, Texas; MEXICO: Coahuila.

##### Notes

MZ-FC

#### Anopliomorpha rinconia

(Casey, 1924)

119965E2-5531-5AB4-9AB8-1466502A1279

##### Materials

- **Type status:** Other material. **Occurrence:** recordedBy: Marysol Trujano and Uri García; **Location:** country: Mexico; stateProvince: Coahuila; municipality: Cuatro Ciénegas; locality: Río Cañón; verbatimCoordinates: 27°00'33.7"N, 102°04'42.3"W; **Event:** samplingProtocol: Light trap; eventDate: 4-Jul-13; habitat: Desertic shrub; **Record Level:** collectionCode: MZ-FC_CCB22; basisOfRecord: Preserved Specimen

##### Ecological interactions

###### Host of

*Quercus grisea* Liebmann (Fagaceae). (Monné 2020)

##### Distribution

USA: Arizona; MEXICO: Baja California Norte, Coahuila (new state record), Oaxaca, Sinaloa, Sonora.

##### Notes

MZ-FC

#### Elaphidionopsis fasciatipennis

Linsley, 1936

9D668555-9306-5A0E-AD0D-D4F4EBC9B1AF

##### Materials

- **Type status:** Other material. **Occurrence:** recordedBy: Marysol Trujano and Uri García; **Location:** country: Mexico; stateProvince: Coahuila; municipality: Cuatro Ciénegas; locality: Rancho PRONATURA; verbatimCoordinates: 26°49'38.9"N, 102°01'24.4"W; **Event:** samplingProtocol: By hand; eventDate: 8-Jul-10; habitat: Desertic shrub; **Record Level:** collectionCode: MZ-FC_CCB23; basisOfRecord: Preserved Specimen

##### Ecological interactions

###### Host of

*Celtis reticulata* Torrey (Cannabaceae), *Dermatophyllum secundiflorum* (Ortega) Gandhi and Reveal (Fabaceae). (Monné 2020)

##### Distribution

USA: Texas; MEXICO: Coahuila.

##### Notes

MZ-FC

#### Neaneflus brevispinus

Chemsak, 1962

6C40EE54-D667-5646-98CE-69252C663279

##### Materials

- **Type status:** Other material. **Occurrence:** recordedBy: Marysol Trujano and Uri García; **Location:** country: Mexico; stateProvince: Coahuila; municipality: Cuatro Ciénegas; locality: Churince; verbatimCoordinates: 26°50'30.3"N, 102°08'9.9"W; **Event:** samplingProtocol: Light trap; eventDate: 20-Aug-12; habitat: Desertic shrub; **Record Level:** collectionCode: MZ-FC_CCB24; basisOfRecord: Preserved Specimen
- **Type status:** Other material. **Occurrence:** recordedBy: Marysol Trujano and Uri García; **Location:** country: Mexico; stateProvince: Coahuila; municipality: Cuatro Ciénegas; locality: Churince; verbatimCoordinates: 26°50'30.3"N, 102°08'9.9"W; **Event:** samplingProtocol: Light trap; eventDate: 20-Aug-12; habitat: Desertic shrub; **Record Level:** collectionCode: MZ-FC_CCB25; basisOfRecord: Preserved Specimen

##### Ecological interactions

###### Host of

*Dalea formosa* Torrey (Fabaceae). (Monné 2020)

##### Distribution

USA: California, Texas; MEXICO: Coahuila (new state record), Zacatecas.

##### Notes

MZ-FC

#### Stenosphenus dolosus

Horn, 1885

5E30F217-8E24-5530-B7E8-DAA119887A4B

##### Materials

- **Type status:** Other material. **Occurrence:** recordedBy: Oscar Pérez; **Location:** country: Mexico; stateProvince: Coahuila; municipality: Cuatro Ciénegas; locality: Poza Azul; verbatimCoordinates: 26°55'28.03"N, 102°06'56.46"W; **Event:** samplingProtocol: Sweeping; eventDate: 29-Jul-15; habitat: Desertic shrub; **Record Level:** collectionCode: CNIN_CCB08; basisOfRecord: Preserved Specimen

##### Ecological interactions

###### Host of

*Baccharis halimifolia* Linnaeus (Asteraceae), *Acacia rigidula* Bentham, *Leucaena* sp., *Prosopis juliflora* (Swartz) de Candolle, *Vachellia farnesiana* (Linnaeus) Wight and Arn., *V. rigidula* (Benth.) Seigler and Ebinger (Mimosaceae). (Monné 2020)

##### Distribution

USA: Texas; MEXICO: Coahuila, Nuevo León, San Luis Potosí, Tamaulipas, Veracruz.

##### Notes

CNIN

#### Hesperophanini

062EB564-F041-57E6-AF89-06CC6D338246

#### Haplidus laticeps

Knull, 1941

E83AB1DC-6456-5454-9667-0CAF279C3EE3

##### Materials

- **Type status:** Other material. **Occurrence:** recordedBy: Marysol Trujano and Uri García; **Location:** country: Mexico; stateProvince: Coahuila; municipality: Cuatro Ciénegas; locality: Río Cañón; verbatimCoordinates: 27°00'33.7"N, 102°04'42.3"W; **Event:** samplingProtocol: Light trap; eventDate: 4-Jul-13; habitat: Desertic shrub; **Record Level:** collectionCode: MZ-FC_CCB26; basisOfRecord: Preserved Specimen

##### Ecological interactions

###### Host of

*Acacia constricta* Bentham ex Gray, *Prosopis juliflora* (Swartz) de Candolle (Mimosaceae). (Monné 2020)

##### Distribution

USA: Texas; MEXICO: Chihuahua, Coahuila (new state record).

##### Notes

MZ-FC

#### Methiini

40F4934D-A21E-5B1E-8F64-A0004154F674

#### Methia mormona

Linell, 1897

91496F3C-15AE-52F6-987E-BAE8955FED10

##### Materials

- **Type status:** Other material. **Occurrence:** recordedBy: Marysol Trujano and Uri García; **Location:** country: Mexico; stateProvince: Coahuila; municipality: Cuatro Ciénegas; locality: Río Cañón; verbatimCoordinates: 27°00'33.7"N, 102°04'42.3"W; **Event:** samplingProtocol: Sweeping; eventDate: 4-Jul-13; habitat: Desertic shrub; **Record Level:** collectionCode: MZ-FC_CCB27; basisOfRecord: Preserved Specimen

##### Ecological interactions

###### Host of

*Berberis harrisoniana* Kearney and Peebles (Berberidaceae), *Juniperus deppeana* Steudel (Cupressaceae), *Juglans major* A. A. Heller (Juglandaceae), *Salix* sp. (Salicaceae). (Monné 2020)

##### Distribution

USA: Arizona, Colorado, New Mexico, Utah; MEXICO: Chihuahua, Coahuila (new state record).

##### Notes

MZ-FC

#### Rhopalophorini

5C564519-4900-57FD-BED7-36965ACD1B8D

#### Rhopalophora angustata

Schaeffer, 1905

7B081B64-5D93-5947-B8F2-084A4A8DBC0D

##### Materials

- **Type status:** Other material. **Occurrence:** recordedBy: Cecilia Fernández; **Location:** country: Mexico; stateProvince: Coahuila; municipality: Cuatro Ciénegas; locality: Emilio Bichara; **Event:** samplingProtocol: By hand; eventDate: 25-Sep-16; habitat: Desertic shrub; **Record Level:** collectionCode: CNIN_CCB09; basisOfRecord: Preserved Specimen
- **Type status:** Other material. **Occurrence:** recordedBy: Cecilia Fernández; **Location:** country: Mexico; stateProvince: Coahuila; municipality: Cuatro Ciénegas; locality: Emilio Bichara; **Event:** samplingProtocol: By hand; eventDate: 25-Sep-16; habitat: Desertic shrub; **Record Level:** collectionCode: CNIN_CCB10; basisOfRecord: Preserved Specimen

##### Ecological interactions

###### Host of

*Baccharis neglecta* Nuttall (Asteraceae), *Celtis berlandieri* Klotzsch (Cannabaceae), *Diospyros texana* Scheele (Ebenaceae), *Eysenhardtia polystachya* (Ortega) Sargent, *E. texana* Scheele (Fabaceae), *Pithecellobium flexicaule* (Bentham) Coulter, *P. pallens* (Bentham) Standley (Mimosaceae), *Citrus sinensis* (Linnaeus) Osbeck, *Zanthoxylum fagara* (Linnaeus) Sargent (Rutaceae). (Monné 2020)

##### Distribution

USA: Texas; MEXICO: Coahuila (new state record), Nuevo León, Tamaulipas.

##### Notes

CNIN

#### Tillomorphini

41CA5687-96B6-57B5-9AC7-35A2807BFA1D

#### Euderces basimaculatus

Giesbert and Chemsak, 1997

E98A2EFE-41C7-5420-B7CF-72E8160EA6C9

##### Materials

- **Type status:** Other material. **Occurrence:** recordedBy: Mayra Cervantes; **Location:** country: Mexico; stateProvince: Coahuila; municipality: Cuatro Ciénegas; locality: PRONATURA; **Event:** samplingProtocol: By hand; eventDate: 21-Aug-15; habitat: Desertic shrub; **Record Level:** collectionCode: CNIN_CCB11; basisOfRecord: Preserved Specimen

##### Ecological interactions

###### Host of

*Lysiloma acapulcense* (Kunth.) Benth., *Haematoxylum brasiletto* H. Karst., *Vachellia pennatula* (Schltdl. and Cham.) Seigler and Ebinger (Fabaceae). (Monné 2020)

##### Distribution

MEXICO: Coahuila (new state record), Colima, Durango, Guerrero, Hidalgo, Jalisco, Michoacán, Nayarit, Oaxaca, Puebla.

##### Notes

CNIN

#### Trachyderini

B424030F-7681-5B4B-B1BA-DF2D1424B70B

#### Chemsakiella michelbacheri

(Chemsak, 1984)

622ADB05-5B9E-5948-8BC6-ED955BF075F6

##### Materials

- **Type status:** Other material. **Occurrence:** recordedBy: Mayra Cervantes; **Location:** country: Mexico; stateProvince: Coahuila; municipality: Cuatro Ciénegas; locality: PRONATURA; **Event:** samplingProtocol: By hand; eventDate: 25-Aug-15; habitat: Desertic shrub; **Record Level:** collectionCode: CNIN_CCB12; basisOfRecord: Preserved Specimen

##### Distribution

MEXICO: Coahuila, Tamaulipas, Zacatecas.

##### Notes

CNIN

#### Crossidius mexicanus

Chemsak and Noguera, 1997

14A5DDB2-2200-52B9-8C59-6EB2EDAD99CD

##### Materials

- **Type status:** Other material. **Occurrence:** recordedBy: Ángel Neria; **Location:** country: Mexico; stateProvince: Coahuila; municipality: Cuatro Ciénegas; locality: Churince; **Event:** samplingProtocol: By hand; eventDate: 18-Aug-14; habitat: Desertic shrub; **Record Level:** collectionCode: CNIN_CCB13; basisOfRecord: Preserved Specimen
- **Type status:** Other material. **Occurrence:** recordedBy: Oscar Pérez; **Location:** country: Mexico; stateProvince: Coahuila; municipality: Cuatro Ciénegas; locality: Churince; verbatimCoordinates: 26°50'19.36"N, 102°09'04.38"W; **Event:** samplingProtocol: Sweeping; eventDate: 21-Jul-15; habitat: Desertic shrub; **Record Level:** collectionCode: CNIN_CCB14; basisOfRecord: Preserved Specimen

##### Distribution

MEXICO: Coahuila, Nuevo León, Zacatecas.

##### Notes

CNIN

#### Crossidius suturalis suturalis

LeConte, 1858

D5677DB0-3C83-5025-8C33-52039452D7A3

##### Materials

- **Type status:** Other material. **Occurrence:** recordedBy: Marysol Trujano and Uri García; **Location:** country: Mexico; stateProvince: Coahuila; municipality: Cuatro Ciénegas; locality: Churince; verbatimCoordinates: 26°50'30.3"N, 102°08'9.9"W; **Event:** samplingProtocol: By hand; eventDate: 4-Jul-13; habitat: Desertic shrub; **Record Level:** collectionCode: MZ-FC_CCB28; basisOfRecord: Preserved Specimen
- **Type status:** Other material. **Occurrence:** recordedBy: Marysol Trujano and Uri García; **Location:** country: Mexico; stateProvince: Coahuila; municipality: Cuatro Ciénegas; locality: Churince; verbatimCoordinates: 26°50'30.3"N, 102°08'9.9"W; **Event:** samplingProtocol: By hand; eventDate: 4-Jul-13; habitat: Desertic shrub; **Record Level:** collectionCode: MZ-FC_CCB29; basisOfRecord: Preserved Specimen
- **Type status:** Other material. **Occurrence:** recordedBy: Marysol Trujano and Uri García; **Location:** country: Mexico; stateProvince: Coahuila; municipality: Cuatro Ciénegas; locality: Churince; verbatimCoordinates: 26°50'30.3"N, 102°08'9.9"W; **Event:** samplingProtocol: By hand; eventDate: 4-Jul-13; habitat: Desertic shrub; **Record Level:** collectionCode: MZ-FC_CCB30; basisOfRecord: Preserved Specimen
- **Type status:** Other material. **Occurrence:** recordedBy: Marysol Trujano and Uri García; **Location:** country: Mexico; stateProvince: Coahuila; municipality: Cuatro Ciénegas; locality: Churince; verbatimCoordinates: 26°50'30.3"N, 102°08'9.9"W; **Event:** samplingProtocol: By hand; eventDate: 26-Apr-12; habitat: Desertic shrub; **Record Level:** collectionCode: MZ-FC_CCB31; basisOfRecord: Preserved Specimen
- **Type status:** Other material. **Occurrence:** recordedBy: Marysol Trujano and Uri García; **Location:** country: Mexico; stateProvince: Coahuila; municipality: Cuatro Ciénegas; locality: Rancho Orozco; verbatimCoordinates: 26°52'18.3"N, 102°05'16.8"W; **Event:** samplingProtocol: By hand; eventDate: 29-Oct-13; habitat: Desertic shrub; **Record Level:** collectionCode: MZ-FC_CCB32; basisOfRecord: Preserved Specimen
- **Type status:** Other material. **Occurrence:** recordedBy: Marysol Trujano and Uri García; **Location:** country: Mexico; stateProvince: Coahuila; municipality: Cuatro Ciénegas; locality: Rancho Orozco; verbatimCoordinates: 26°52'18.3"N, 102°05'16.8"W; **Event:** samplingProtocol: By hand; eventDate: 29-Oct-13; habitat: Desertic shrub; **Record Level:** collectionCode: MZ-FC_CCB33; basisOfRecord: Preserved Specimen

##### Ecological interactions

###### Host of

*Chrysothamnus viscidiflorus* (Hooker) Nuttall, *Gutierrezia longifolia* Greene, *G. lucida* Greene, *G. wrightii* A. Gray, *Haplopappus hartwegi* (Gray) Blake, *H. pluriflorus* (Gray) Hall, *Xanthocephalum glutinosum* (Sprengel) Shinners, *X. sarothrae* (Pursh) Shinners (Asteraceae). (Monné 2020)

##### Distribution

USA: Arizona, New Mexico, Texas; MEXICO: Chihuahua, Coahuila (new state record), Sonora.

##### Notes

MZ-FC

#### Elytroleptus divisus

(LeConte, 1884)

909040BA-E74E-580E-B9D8-21750A60E94D

##### Materials

- **Type status:** Other material. **Occurrence:** recordedBy: Oscar Pérez; **Location:** country: Mexico; stateProvince: Coahuila; municipality: Cuatro Ciénegas; locality: Churince; verbatimCoordinates: 26°50'19.36"N, 102°09'04.38"W; **Event:** samplingProtocol: Sweeping; eventDate: 21-Jul-15; habitat: Desertic shrub; **Record Level:** collectionCode: CNIN_CCB15; basisOfRecord: Preserved Specimen

##### Distribution

USA: Texas; MEXICO: Coahuila, Nuevo León, Querétaro, San Luis Potosí, Tamaulipas.

##### Notes

CNIN

#### Neocrossidius trivittatus

(Bates, 1880)

D564C25A-EC77-5E56-9240-4DDA64ED9A68

##### Materials

- **Type status:** Other material. **Occurrence:** recordedBy: Marysol Trujano and Uri García; **Location:** country: Mexico; stateProvince: Coahuila; municipality: Cuatro Ciénegas; locality: San José del Anteojo; verbatimCoordinates: 26°58'11.5"N, 102°07'26.4"W; **Event:** samplingProtocol: Sweeping; eventDate: 23-May-12; habitat: Desertic shrub; **Record Level:** collectionCode: MZ-FC_CCB34; basisOfRecord: Preserved Specimen
- **Type status:** Other material. **Occurrence:** recordedBy: Marysol Trujano and Uri García; **Location:** country: Mexico; stateProvince: Coahuila; municipality: Cuatro Ciénegas; locality: San José del Anteojo; verbatimCoordinates: 26°58'11.5"N, 102°07'26.4"W; **Event:** samplingProtocol: Sweeping; eventDate: 23-May-12; habitat: Desertic shrub; **Record Level:** collectionCode: MZ-FC_CCB35; basisOfRecord: Preserved Specimen

##### Distribution

MEXICO: Chihuahua, Ciudad de México, Coahuila, Durango, Michoacán, Morelos.

##### Notes

MZ-FC

#### Parevander hovorei

Giesbert, 1984

62BD48DF-6A59-53FB-B836-20BC937102C4

##### Materials

- **Type status:** Other material. **Occurrence:** recordedBy: Marysol Trujano and Uri García; **Location:** country: Mexico; stateProvince: Coahuila; municipality: Cuatro Ciénegas; locality: Antiguos Mineros; verbatimCoordinates: 26°46'57.7"N, 102°00'20.2"W; **Event:** samplingProtocol: By hand; eventDate: 8-Jul-10; habitat: Desertic shrub; **Record Level:** collectionCode: MZ-FC_CCB36; basisOfRecord: Preserved Specimen
- **Type status:** Other material. **Occurrence:** recordedBy: Marysol Trujano and Uri García; **Location:** country: Mexico; stateProvince: Coahuila; municipality: Cuatro Ciénegas; locality: Antiguos Mineros; verbatimCoordinates: 26°46'57.7"N, 102°00'20.2"W; **Event:** samplingProtocol: By hand; eventDate: 8-Jul-10; habitat: Desertic shrub; **Record Level:** collectionCode: MZ-FC_CCB37; basisOfRecord: Preserved Specimen
- **Type status:** Other material. **Occurrence:** recordedBy: Marysol Trujano and Uri García; **Location:** country: Mexico; stateProvince: Coahuila; municipality: Cuatro Ciénegas; locality: Antiguos Mineros; verbatimCoordinates: 26°46'57.7"N, 102°00'20.2"W; **Event:** samplingProtocol: By hand; eventDate: 8-Jul-10; habitat: Desertic shrub; **Record Level:** collectionCode: MZ-FC_CCB38; basisOfRecord: Preserved Specimen

##### Ecological interactions

###### Host of

*Helianthus* sp., *Verbesina* sp., *Viguiera* sp. (Asteraceae). (Monné 2020)

##### Distribution

USA: Texas; MEXICO: Coahuila, Hidalgo, Nuevo León, San Luis Potosí, Tamaulipas.

##### Notes

MZ-FC

#### Plionoma suturalis

(LeConte, 1858)

36F891B9-F03B-5F0F-85B2-BF78BE30CED4

##### Materials

- **Type status:** Other material. **Occurrence:** recordedBy: Marysol Trujano and Uri García; **Location:** country: Mexico; stateProvince: Coahuila; municipality: Cuatro Ciénegas; locality: Río Cañón; verbatimCoordinates: 27°00'33.7"N, 102°04'42.3"W; **Event:** samplingProtocol: Sweeping; eventDate: 31-May-13; habitat: Desertic shrub; **Record Level:** collectionCode: MZ-FC_CCB39; basisOfRecord: Preserved Specimen
- **Type status:** Other material. **Occurrence:** recordedBy: Marysol Trujano and Uri García; **Location:** country: Mexico; stateProvince: Coahuila; municipality: Cuatro Ciénegas; locality: Churince; verbatimCoordinates: 26°50'30.3"N, 102°08'9.9"W; **Event:** samplingProtocol: Sweeping; eventDate: 30-Apr-13; habitat: Desertic shrub; **Record Level:** collectionCode: MZ-FC_CCB40; basisOfRecord: Preserved Specimen

##### Ecological interactions

###### Host of

*Prosopis juliflora* (Swartz) de Candolle, *Vachellia farnesiana* (Linnaeus) Wight and Arn. (Mimosaceae). (Monné 2020)

##### Distribution

USA: California, Texas; MEXICO: Baja California Norte, Coahuila (new state record), Sonora.

##### Notes

MZ-FC

#### Sphaenothecus bilineatus

(Gory in Guérin-Méneville, 1831)

ECF09E31-DC62-58BB-96E7-91EE86EFB1E2

##### Materials

- **Type status:** Other material. **Occurrence:** recordedBy: Marysol Trujano and Uri García; **Location:** country: Mexico; stateProvince: Coahuila; municipality: Cuatro Ciénegas; locality: San José del Anteojo; verbatimCoordinates: 26°58'11.5"N, 102°07'26.4"W; **Event:** samplingProtocol: Sweeping; eventDate: 23-May-12; habitat: Desertic shrub; **Record Level:** collectionCode: MZ-FC_CCB41; basisOfRecord: Preserved Specimen

##### Ecological interactions

###### Host of

*Baccharis* sp. (Asteraceae), *Cassia* sp. (Caesalpiniaceae), *Leucaena pulverulenta* (Schlechtendal) Bentham, *Prosopis juliflora* (Swartz) de Candolle, *Vachellia farnesiana* (Linnaeus) Wight and Arn. (Mimosaceae), *Ficus* sp. (Moraceae), *Coffea arabica* Linnaeus (Rubiaceae), *Ulmus crassifolia* Nuttall (Ulmaceae). (Monné 2020)

##### Distribution

USA: California, Texas; MEXICO: Baja California, Chiapas, Coahuila, Hidalgo, Jalisco, Morelos, Oaxaca, Sonora, Tamaulipas; HONDURAS; NICARAGUA.

##### Notes

MZ-FC

#### Stenaspis solitaria

(Say, 1824)

7C186538-69F3-52C7-82F8-3DEDD45C03BD

##### Materials

- **Type status:** Other material. **Occurrence:** recordedBy: Marysol Trujano and Uri García; **Location:** country: Mexico; stateProvince: Coahuila; municipality: Cuatro Ciénegas; locality: Rancho Orozco; verbatimCoordinates: 26°52'18.3"N, 102°05'16.8"W; **Event:** samplingProtocol: By hand; eventDate: 3-Jul-13; habitat: Desertic shrub; **Record Level:** collectionCode: MZ-FC_CCB42; basisOfRecord: Preserved Specimen
- **Type status:** Other material. **Occurrence:** recordedBy: Marysol Trujano and Uri García; **Location:** country: Mexico; stateProvince: Coahuila; municipality: Cuatro Ciénegas; locality: Rancho Orozco; verbatimCoordinates: 26°52'18.3"N, 102°05'16.8"W; **Event:** samplingProtocol: By hand; eventDate: 3-Jul-13; habitat: Desertic shrub; **Record Level:** collectionCode: MZ-FC_CCB43; basisOfRecord: Preserved Specimen

##### Ecological interactions

###### Host of

*Acacia berlandieri* Bentham, *Prosopis juliflora* (Swartz) de Candolle (Mimosaceae). (Monné 2020)

##### Distribution

USA: Arizona, California, Texas; MEXICO: Baja California Norte, Coahuila (new state record), Durango, Sonora.

##### Notes

MZ-FC

#### Tylosis jimenezi

Dugés, 1879

94E43A29-A724-5A38-8B93-090827A5FA46

##### Materials

- **Type status:** Other material. **Occurrence:** recordedBy: Marysol Trujano and Uri García; **Location:** country: Mexico; stateProvince: Coahuila; municipality: Cuatro Ciénegas; locality: Rancho Orozco; verbatimCoordinates: 26°52'18.3"N, 102°05'16.8"W; **Event:** samplingProtocol: Sweeping; eventDate: 3-Jul-13; habitat: Desertic shrub; **Record Level:** collectionCode: MZ-FC_CCB44; basisOfRecord: Preserved Specimen
- **Type status:** Other material. **Occurrence:** recordedBy: Marysol Trujano and Uri García; **Location:** country: Mexico; stateProvince: Coahuila; municipality: Cuatro Ciénegas; locality: Rancho Orozco; verbatimCoordinates: 26°52'18.3"N, 102°05'16.8"W; **Event:** samplingProtocol: Sweeping; eventDate: 3-Jul-13; habitat: Desertic shrub; **Record Level:** collectionCode: MZ-FC_CCB45; basisOfRecord: Preserved Specimen
- **Type status:** Other material. **Occurrence:** recordedBy: Marysol Trujano and Uri García; **Location:** country: Mexico; stateProvince: Coahuila; municipality: Cuatro Ciénegas; locality: Rancho Orozco; verbatimCoordinates: 26°52'18.3"N, 102°05'16.8"W; **Event:** samplingProtocol: Sweeping; eventDate: 3-Jul-13; habitat: Desertic shrub; **Record Level:** collectionCode: MZ-FC_CCB46; basisOfRecord: Preserved Specimen

##### Ecological interactions

###### Host of

*Sphaeralcea* sp. (Malvaceae). (Monné 2020)

##### Distribution

USA: Texas; MEXICO: Ciudad de México, Coahuila, Durango, Guanajuato.

##### Notes

MZ-FC

#### Lamiinae

4CCD2C1C-5270-528A-9CA3-19D8DA6ACA10

#### Acanthocinini

DDE93E9C-4B97-5E4C-B46E-E3523BAEF08F

#### Moneilema armatum

LeConte, 1853

E74773D3-93A0-5421-8408-A1DFED3F6A86

##### Materials

- **Type status:** Other material. **Occurrence:** recordedBy: Marysol Trujano and Uri García; **Location:** country: Mexico; stateProvince: Coahuila; municipality: Cuatro Ciénegas; locality: Churince; verbatimCoordinates: 26°50'30.3"N, 102°08'9.9"W; **Event:** samplingProtocol: By hand; eventDate: 6-Jun-10; habitat: Desertic shrub; **Record Level:** collectionCode: MZ-FC_CCB47; basisOfRecord: Preserved Specimen

##### Ecological interactions

###### Host of

*Astrophytum asterias* (Zuccarini) Lemaire, *Homalocephala texensis* Britton and Rose, *Marginatocereus marginatus* (de Candolle) Beckeberg, *Opuntia arborescens* Engelmann, *O. arbuscula* Engelmann, *O. engelmanni* Salm-Dyck, *O. imbricata* de Candolle, *O. leptocaulis* de Candolle, *O. lindheimeri* Engelmann, *O. macrocentra* Engelmann, *O. megacantha* Salm-Dyck, *O. robusta* Wendland, *O. spinosior* Toumey, *O. violacea* Engelmann, *Platyopuntia* sp. (Cactaceae). (Monné 2020)

##### Distribution

USA: Colorado, Kansas; MEXICO: Coahuila, Tamaulipas.

##### Notes

MZ-FC

#### Onciderini

07D9E1C7-CC0B-587A-BB85-8D8B54249865

#### Oncideres rhodosticta

Bates, 1885

635FFD1F-8EF9-5F66-9523-14DC8CAD11F2

##### Materials

- **Type status:** Other material. **Occurrence:** recordedBy: Marysol Trujano and Uri García; **Location:** country: Mexico; stateProvince: Coahuila; municipality: Cuatro Ciénegas; locality: Poza Azul; verbatimCoordinates: 26°55'21.2"N, 102°72'3.6"W; **Event:** samplingProtocol: Light trap; eventDate: 24-Aug-12; habitat: Desertic shrub; **Record Level:** collectionCode: MZ-FC_CCB48; basisOfRecord: Preserved Specimen
- **Type status:** Other material. **Occurrence:** recordedBy: Marysol Trujano and Uri García; **Location:** country: Mexico; stateProvince: Coahuila; municipality: Cuatro Ciénegas; locality: Poza Azul; verbatimCoordinates: 26°55'21.2"N, 102°72'3.6"W; **Event:** samplingProtocol: Light trap; eventDate: 24-Aug-12; habitat: Desertic shrub; **Record Level:** collectionCode: MZ-FC_CCB49; basisOfRecord: Preserved Specimen

##### Ecological interactions

###### Host of

*Parkinsonia aculeata* Linnaeus (Caesalpiniaceae), *Casuarina cuninghamiana* Miquel (Casuarinaceae), *Sarcobatus vermiculatus* (Hooker) Torrey (Chenopodiaceae), *Acacia greggii* A.Gray, *Mimosa* sp., *Pithecellobium flexicaule* (Bentham) Coulter, *Prosopis juliflora* (Swartz) de Candolle, *P. glandulosa* Torrey, *Vachellia farnesiana* (Linnaeus) Wight. and Arn. (Mimosaceae). (Monné 2020)

##### Distribution

USA: Arizona, New Mexico, Texas; MEXICO: Baja California Norte, Chihuahua, Coahuila, Durango, Sonora, Tamaulipas.

##### Notes

MZ-FC

## Analysis

As this study is the first species list of Cerambycidae fauna in a Mexican desert environment, it greatly increases the knowledge of this group in Mexico. We recorded 37 species from 32 genera and identified 13 tribes from four subfamilies (Prioninae, Lepturinae, Cerambycinae and Lamiinae) Table 1. The subfamily Cerambycinae had the greatest number of species at 31 (84% of the total). The other subfamilies only registered two species each. This revealed a pattern similar to that of other habitats such as tropical dry forest (e.g. Toledo et al. 2002, Noguera et al. 2012), but in this case, the results were even more pronounced.

## Discussion

Despite the fact that Mexico has extensive area of deserts, faunistic studies relating to Cerambycidae have previously not been conducted in this or similar xerophilous habitats. In Mexico, the family Cerambycidae has been studied mainly in tropical dry forest (e.g. Chemsak and Noguera 1993, Noguera et al. 2012). Only sporadic records from desertic areas have been reported (Bezark 2020).

MacKay et al. (1987) recorded 44 species from the northern region of the Chihuahuan desert near Las Cruces, New Mexico, USA. Compared to our study, they obtained the same species distribution in subfamilies. Cerambycinae were extremely abundant, followed by Prioninae and Lamiinae, both being well represented. Only one species of Lepturinae was recorded. MacKay et al. (1987) argue that Lepturinae species depend on moisture for development, hence their species richness in arid environments is poor. On the another hand, Cerambycinae presents the greatest number of genera with diurnal habits (Linsley 1959). In the case of CCB, the genera of Cerambycinae were more common in daytime activity. This is presumably due to different types of adult feeding which is anthophilous in several genera of Cerambycinae (Švácha and Lawrence 2014).

Of the species recorded in this study, *Meloemorpha aliena* (Bates), *Knulliana cincta cincta* (Drury), *Megacyllene antennata* (White), *Aneflomorpha rectilinea rectilinea* Casey, *Anopliomorpha rinconia* (Casey), *Neaneflus brevispinus* Chemsak, *Haplidus laticeps* Knull, *Methia mormona* Linell, *Rhopalophora angustata* Schaeffer, *Euderces basimaculatus* Giesbert & Chemsak, *Crossidius suturalis suturalis* LeConte, *Plionoma suturalis* (LeConte) and *Stenaspis solitaria* (Say) are new records for the state of Coahuila. *Typocerus sinuatus* (Newman), *Neoclytus mucronatus mucronatus* (Fabricius) and *Aneflomorpha werneri* Chemsak are reported for the first time in Mexico. These three species have been previously recorded in the southern United States (Texas).

Possibly due in part to extensive collecting efforts, the states of Veracruz (406), Oaxaca (343), Jalisco (327) and Chiapas (315) (Noguera 2014) have the highest species records of Cerambycidae (numbers in parenthesis indicate the numbers of species recorded from the corresponding state). Noguera (2014) indicates the species richness from Coahuila at 24 species. Nevertheless, Bezark (2020) in the electronic catalogue of Cerambycidae recorded 43 species from Coahuila. Our work represents a considerable contribution and updated inventory of Mexican Cerambycidae in the Coahuila region. It presents new distributional records that represent a 41% increase in species richness. It is clear that it is necessary to continue exploring more regions of Mexico and publishing updated Cerambycidae distribution data to complete our knowledge, not only of desert environments, but also of other Mexican ecosystems present in the country.

The distribution of species that were recorded in CCB shows a mixture between cerambycid species with distributional records in United States and Mexico (Bezark 2020). Many of these species have diurnal habits and an affinity for desert environments. This is useful for understanding the distributional patterns and evolutionary history of cerambycid beetles. The ecological factors and historical events from the *Altiplano Mexicano* are relevant for determining the geographic distribution of insect species from CCB (Álvarez and Ojeda 2019). Additionally, the presence of host plants in these desert ecosystems is fundamental to their existence in these environments (Pinkava 2016).

The CCB is a unique region with different components from Neotropical and Nearctic biogeographic provinces. This makes it an essential crossroads to understand the evolution of several faunal groups (Álvarez and Ojeda 2019). This work represents the first species list of Cerambycidae in a Mexican desert (CCB, Chihuahuan desert) and Coahuila. Cerambycids provide important ecological services for desert ecosystems. They act as pollinators (MacKay et al. 1987), active agents of decomposition and recyclers of organic matter. As ecosystem agents, these beetles are indispensable for the health and permanency of CCB environments.

## Supplementary Material

39148492-4E32-51ED-9426-B8F2F49F0F95 10.3897/BDJ.8.e54495.suppl1 Supplementary material 1 Checklist_Taxon_CCB Data type Taxon information and ocurrence information File: oo_428407.xlsx https://binary.pensoft.net/file/428407 Pérez-Flores, O.

XML Treatment for Prioninae

XML Treatment for Prionini

XML Treatment for Derobrachus hovorei

XML Treatment for Prionus (Prionus) poultoni

XML Treatment for Lepturinae

XML Treatment for Lepturini

XML Treatment for Meloemorpha aliena

XML Treatment for Typocerus sinuatus

XML Treatment for Cerambycinae

XML Treatment for Bothriospilini

XML Treatment for Knulliana cincta cincta

XML Treatment for Clytini

XML Treatment for Megacyllene antennata

XML Treatment for Neoclytus mucronatus mucronatus

XML Treatment for Placosternus erythropus

XML Treatment for Tanyochraethes hololeucus

XML Treatment for Eburiini

XML Treatment for Eburia juanitae

XML Treatment for Eburia maccartyi

XML Treatment for Eburia paraegrota

XML Treatment for Elaphidiini

XML Treatment for Aneflus (Aneflus) obscurus

XML Treatment for Aneflus (Aneflus) prolixus insoletus

XML Treatment for Aneflomorpha rectilinea rectilinea

XML Treatment for Aneflomorpha werneri

XML Treatment for Anelaphus moestus moestus

XML Treatment for Anopliomorpha rinconia

XML Treatment for Elaphidionopsis fasciatipennis

XML Treatment for Neaneflus brevispinus

XML Treatment for Stenosphenus dolosus

XML Treatment for Hesperophanini

XML Treatment for Haplidus laticeps

XML Treatment for Methiini

XML Treatment for Methia mormona

XML Treatment for Rhopalophorini

XML Treatment for Rhopalophora angustata

XML Treatment for Tillomorphini

XML Treatment for Euderces basimaculatus

XML Treatment for Trachyderini

XML Treatment for Chemsakiella michelbacheri

XML Treatment for Crossidius mexicanus

XML Treatment for Crossidius suturalis suturalis

XML Treatment for Elytroleptus divisus

XML Treatment for Neocrossidius trivittatus

XML Treatment for Parevander hovorei

XML Treatment for Plionoma suturalis

XML Treatment for Sphaenothecus bilineatus

XML Treatment for Stenaspis solitaria

XML Treatment for Tylosis jimenezi

XML Treatment for Lamiinae

XML Treatment for Acanthocinini

XML Treatment for Moneilema armatum

XML Treatment for Onciderini

XML Treatment for Oncideres rhodosticta

## Figures and Tables

**Table 1.** Number of tribes, genera and species of the cerambycids from Cuatro Ciénegas Basin

Subfamily	No. of tribes	No. of genera	No. of species
Prioninae	1	2	2
Lepturinae	1	2	2
Cerambycinae	9	26	31
Lamiinae	2	2	2
Total	13	32	37
